# Local Structure and Coordination Define Adsorption in a Model Ir_1_/Fe_3_O_4_ Single‐Atom Catalyst

**DOI:** 10.1002/anie.201907536

**Published:** 2019-08-19

**Authors:** Zdenek Jakub, Jan Hulva, Matthias Meier, Roland Bliem, Florian Kraushofer, Martin Setvin, Michael Schmid, Ulrike Diebold, Cesare Franchini, Gareth S. Parkinson

**Affiliations:** ^1^ Institute of Applied Physics TU Wien Wiedner Hauptstr. 8–10/134 1040 Vienna Austria; ^2^ Current Address: Advanced Research Center for Nanolithography (ARCNL) 1090 BA Amsterdam The Netherlands; ^3^ Center for Computational Materials Science Faculty of Physics University of Vienna 1090 Vienna Austria

**Keywords:** adsorption, heterogeneous catalysis, scanning probe microscopy, single-atom catalysis

## Abstract

Single‐atom catalysts (SACs) bridge homo‐ and heterogeneous catalysis because the active site is a metal atom coordinated to surface ligands. The local binding environment of the atom should thus strongly influence how reactants adsorb. Now, atomically resolved scanning‐probe microscopy, X‐ray photoelectron spectroscopy, temperature‐programmed desorption, and DFT are used to study how CO binds at different Ir_1_ sites on a precisely defined Fe_3_O_4_(001) support. The two‐ and five‐fold‐coordinated Ir adatoms bind CO more strongly than metallic Ir, and adopt structures consistent with square‐planar Ir^I^ and octahedral Ir^III^ complexes, respectively. Ir incorporates into the subsurface already at 450 K, becoming inactive for adsorption. Above 900 K, the Ir adatoms agglomerate to form nanoparticles encapsulated by iron oxide. These results demonstrate the link between SAC systems and coordination complexes, and that incorporation into the support is an important deactivation mechanism.

## Graphical abstract

**The coordination of a single atom** to the oxide support has dramatic consequences on its ability to adsorb carbon monoxide. The observed motifs can be rationalized using simple arguments from coordination chemistry, confirming the hypothesis that single‐atom catalysts have much in common with organometallic complexes used in homogeneous catalysis.

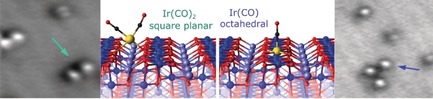

## Introduction

The field of single‐atom catalysis (SAC)^1^ arose as the ultimate extension of attempts to reduce the precious‐metal content of supported heterogeneous catalysts. While it now seems established that supported metal adatoms can catalyze heterogeneous and electrochemical reactions, it is increasingly clear that the properties of single‐atom catalysts (SACs) differ significantly from those of supported metal nanoparticles.1e, 2 This is because stable adatoms must derive their stability from chemical bonds to the support lattice, which modifies their electronic structure, and thus their interaction with reactants.1b Reaction mechanisms can also differ in the single‐atom limit owing to the lack of nearest‐neighbor metal sites (for example, two neighboring sites are required to dissociate O_2_), making it difficult to predict which metal/support combination might be the best choice for a particular reaction. Theoretical screening studies suggest several Me_1_/FeO_*x*_ systems will outperform Pt for CO oxidation,^3^ but which metal adatom yields the best performance depends on the reaction mechanism that is assumed. Moreover, it is not clear whether the assumed adsorption site exists in real SAC systems, which are complex, often inhomogeneous, and difficult to characterize.

In recent years there has been growing excitement that SAC might allow to bridge hetero‐ and homogeneous catalysis,^4^ combining the high throughput and reusability of the former with the exquisite selectivity typical of the latter. Researchers have begun to synthesize SACs that mimic aspects of well‐known homogeneous catalysts, and there has been early success heterogenizing reactions typically performed in solution.4b, 5 The analogy to homogeneous catalysis suggests that the catalytic properties should depend strongly on the local coordination environment of the metal, since the bonds to the support are akin to ligands. There is some evidence of this effect,^6^ but most fundamental work has focused on Pt‐based SACs rather than metals such as Rh and Ir.

In this work, we study how Ir_1_ species bind to a precisely defined Fe_3_O_4_(001) support,^7^ and correlate the different sites with the ability of the model catalyst to adsorb CO. This system was selected because Ir_1_/FeO_*x*_ catalysts have already been shown to be active for both CO oxidation^3^ and the water‐gas shift reaction,^8^ where CO is a reactant, and because Ir‐based coordination complexes are common in homogeneous catalysis. Moreover, IrO_2_ is an important catalyst for water oxidation,^9^ and an efficient Ir‐based SAC might present a way to reduce the amount of Ir required for this important reaction. Finally, the interaction of CO with metallic Ir is well‐characterized,^10^ allowing direct comparisons to be made. Our work shows that Ir atoms can take three different geometries with two‐, five‐, and six‐fold coordination to the lattice oxygen. The two‐ and five‐fold sites bind CO more strongly than metallic Ir, and form structures consistent with Ir^I^ and Ir^III^ coordination complexes. The six‐fold site is energetically preferred, but is subsurface and unable to adsorb CO.

## Results and Discussion

**Figure 1:**
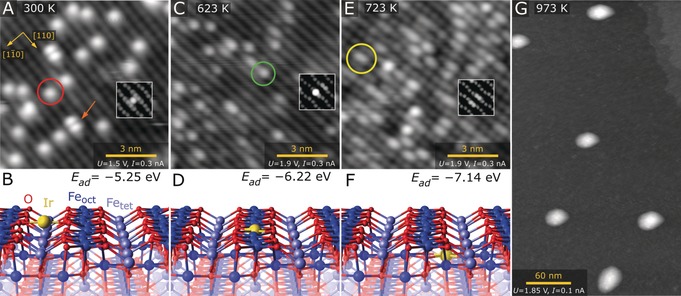
Determining the local structure of the Ir_1_/Fe_3_O_4_(001) model catalyst using room‐temperature STM and DFT. A) Ir_1_ atoms evaporated directly onto the Fe_3_O_4_(001) surface at 300 K are imaged as bright protrusions between the Fe rows of the support (red circle in STM image). Double protrusions are metastable Ir_2_ dimers (orange arrow). B) DFT‐derived minimum‐energy structure of the two‐fold‐coordinated Ir adatom on Fe_3_O_4_(001). An STM simulation based on this structure is shown as an inset in (A). C) After annealing at 623 K, Ir atoms appear as bright protrusions within the Fe row in STM images (green circle). D) DFT‐derived minimum‐energy structure of the five‐fold‐coordinated Ir atom incorporated within the Fe_3_O_4_(001) surface, with the corresponding STM simulation shown as an inset in (C). E) At 723 K, some of the bright protrusions within the row are replaced by extended bright protrusions in STM (yellow circle). Some small irregular clusters are also observed. F) DFT‐derived minimum‐energy structure of the six‐fold‐coordinated Ir adatom incorporated in the subsurface layer of Fe_3_O_4_(001). An STM simulation based on this structure is shown as an inset in (E). G) Annealing at 973 K leads to formation of metallic Ir clusters with an apparent height of about 3 nm.

Figure 1 A shows an STM image of the UHV‐prepared Fe_3_O_4_(001)‐($\sqrt{2}$ ×$\sqrt{2}$ )R 45° surface after 0.13 ML Ir was thermally evaporated onto the sample at room temperature. The bright rows running in the [110] direction are due to the five‐fold coordinated Fe atoms of the support (blue in the DFT model in Figure 1 B), which exhibits the subsurface cation vacancy (SCV) termination.^7^ Note that surface oxygen atoms (red in the model) are not imaged because they possess no density of states (DOS) in the vicinity of the Fermi level (*E*_F_), but their positions are precisely known from quantitative structural measurements and theoretical computations.^7^ Isolated Ir_1_ adatoms appear as bright protrusions between the rows, as has been observed previously for a variety of other metals on this surface.^11^ The adsorption site is essentially where the next tetrahedrally coordinated Fe cation would reside in the spinel structure,11d, 11e suggesting a two‐fold coordination to the substrate. This is in line with our DFT calculations (optB88‐DF,^12^ *U*_eff_=3.61 eV), which find a strong binding energy (−5.2 eV with respect to a single Ir atom in the gas phase) and small positive Bader charge of 0.5 e for an Ir adatom in this position. A full listing of the Bader charges and magnetic moments for all of the configurations discussed here is given in the Supporting Information, Table S1. An STM simulation of the structure shown in Figure 1 B (inset in Figure 1 A) is consistent with the measured data. Some double protrusions are also observed (red arrow), which we attribute to Ir_2_ dimers, but these disappear already upon mild annealing in experiment. This suggests that Ir dimers are unstable with respect to Ir adatoms, similarly to previous observations of Ag and Pt on this surface.11a, 11c

When the model SAC is heated to 623 K (Figure 1 C), the two‐fold Ir_1_ adatoms disappear, and we instead observe protrusions located within the surface Fe rows. The most common species are isolated bright features (green circle), which we identify as Ir atoms substituting five‐fold Fe atoms in the surface layer. Our DFT calculations (Figure 1 D) show that this site is 1 eV more stable than the two‐fold site, and that the Ir is more oxidized in this configuration (Bader charge 1.4 e). In the model shown, the Fe atom displaced by Ir occupies one of the octahedral vacancies in the second Fe−O layer. This change in the subsurface cation ordering would manifest as an elongated, less bright feature close to the Ir‐related protrusion (see accompanying STM simulation; inset in Figure 1 C). While this is observed in some cases, we note that Fe exchange with the bulk is already facile at this temperature,^13^ so it is also possible that the excess Fe diffuses into deeper layers. In this case, a single bright protrusion in the row would be observed. A phase diagram constructed using DFT and atomistic thermodynamics^14^ (Supporting Information, Figure S1) suggests that these two configurations are similarly stable in the ranges of O_2_ chemical potential considered here.

When the sample is heated to 723 K (Figure 1 E), the number of five‐fold Ir atoms decreases, and we observe an increase in the bright protrusions elongated in the [110] direction (yellow circle). Our DFT calculations suggest that these features are due to Ir adatoms in six‐fold coordinated sites in the second Fe−O layer, which is a further 1 eV more stable than the five‐fold site in the surface layer. This subsurface Ir has a similar Bader charge of 1.4 e. In the corresponding STM simulations, the defect appears as either a single or double elongated protrusion, depending on whether the displaced Fe disappears to the bulk or remains in the second Fe−O layer. In this case, the ab initio thermodynamics (Supporting Information, Figure S1) suggests that the excess Fe will remain in the subsurface, so mostly double protrusions are expected. It is important to note, however, that the calculations omit entropic effects, which will favor diffusion of the excess Fe into the sample bulk. In this case, single elongated protrusions are expected.

Moving the Ir atom from the second Fe−O layer to deeper layers costs energy (Supporting Information, Figure S2). This suggests that the size of the Ir adatom can be more easily accommodated in the immediate subsurface than in the bulk, as the surface atoms above the Ir are free to relax. In the final step of the experiments, the sample was heated to 973 K. This results in large Ir clusters spread widely over the surface.

**Figure 2:**
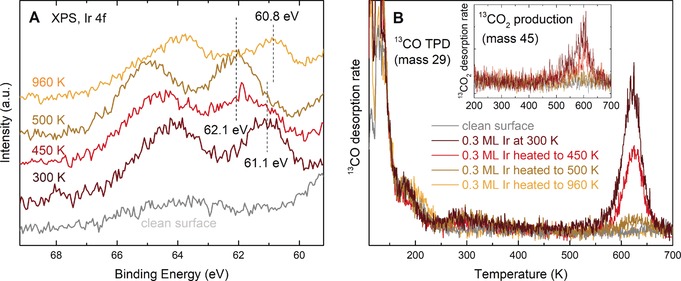
Experimental characterization of the Ir_1_/Fe_3_O_4_ model catalyst by X‐ray photoelectron spectroscopy (XPS) and temperature‐programmed desorption (TPD). A) In XPS, the Ir 4f peaks shift to higher binding energy as the sample is heated, consistent with the occupation of more stable, higher‐coordination sites predicted by DFT. At 960 K, the peak shifts back to the position of metallic Ir owing to the formation of metallic Ir nanoparticles. B) ^13^CO‐TPD shows a single peak at 610 K due to desorption from Ir (peaks below 300 K are due to the Fe_3_O_4_(001) support^17^). This peak decreases in intensity when the sample is heated prior to CO adsorption. No CO desorption is observed from the Ir nanoparticles formed when the sample is heated to 960 K.

XPS measurements of the room‐temperature‐prepared Ir_1_/Fe_3_O_4_(001) surface (Figure 2 A) reveal a single Ir 4f 5/2 peak with binding energy of 61.1 eV for the two‐fold coordinated Ir species. Heating the sample to 450 K for 7 minutes leads to a broad Ir 4f spectrum with a peak at 62.0 eV, although a shoulder remains at 61.1 eV suggesting the transition to the more oxidized five‐ and six‐fold‐coordinated states occurs slowly at this temperature. At 500 K, the spectrum exhibits a single peak at 62.1 eV, close to the 61.8 eV reported for octahedrally coordinated Ir^4+^ in IrO_2_(110).^15^ When the sample is annealed at 960 K, the Ir 4f signal shifts to 60.8 eV, consistent with the formation of metallic Ir nanoparticles (Ir in a bulk metal environment has a 4f 5/2 peak at 60.7 eV^14^). It should be noted that the sample temperatures in the XPS/TPD setup were measured by a thermocouple spot‐welded directly on the sample mount,^16^ and thus are more reliable than those measured in the STM chamber, where the thermocouple is placed further away from the sample plate on the annealing stage. Additionally, the sample mount was getting loose during STM experiments, therefore we estimate the annealing temperatures to be up to 100 K lower than indicated by the thermocouple readout in the STM chamber.

To study how the different coordination environments affect the reactivity of the model catalyst we performed TPD experiments using isotopically labelled ^13^CO as a probe molecule (Figure 2 B). First, 0.3 ML Ir was deposited on the freshly prepared Fe_3_O_4_(001) surface, and the model catalyst with two‐fold Ir exposed to 1 ML ^13^CO using a calibrated molecular beam source^16^ at 100 K. The sample was then heated with a temperature ramp of 1 K s^−1^ and the desorbing species monitored by a mass spectrometer. We have previously shown that CO interacts weakly with the as‐prepared Fe_3_O_4_(001) surface,^17^ desorbing in two peaks below 120 K. The addition of the two‐fold‐coordinated Ir adatoms leads to a new desorption feature at about 610 K, well above the temperature where CO desorbs from metallic Ir surfaces (500–560 K).^10^ Using the Redhead equation with the maximum prefactor at 610 K (3×10^18^ s^−1^), that is, assuming no entropic contribution due to translational or rotational degrees of freedom on the surface, we estimate a CO desorption energy of 2.4±0.1 eV. Apart from a significant ^13^CO desorption signal, we observe a small, broad ^13^CO_2_ peak at about 600 K (see inset in Figure 2 B). Above 600 K, no adsorbed CO is detectable by XPS, and the Ir 4f signal appears at 62.1 eV (Supporting Information, Figure S3).

To quantify the CO_2_ production and confirm that it originates from a Mars–van Krevelen type‐mechanism, we annealed the surface in isotopically labelled ^18^O at 740 K for 3 h. This creates an isotopically enriched Fe_3_^18^O_4_(001) surface as judged by low energy ion scattering (LEIS; Supporting Information, Figure S4). Performing the ^13^CO‐TPD experiment on a surface with three different Ir coverages (0.14, 0.28, 0.56 ML; Supporting Information, Figure S5), we observe a signal in mass 47 around 590 K that increases with increasing Ir coverage. This corresponds to CO_2_ with labeled carbon and one isotopically labelled oxygen atom from the support that is, ^13^C^16^O^18^O. Comparing the peak area with that of CO and correcting for mass spectrometer sensitivity, we estimate that 12–15 % of the Ir‐adsorbed CO is oxidized during the TPD experiment.

When the samples were pre‐annealed at 450 K prior to CO adsorption (to convert the two‐fold Ir into five‐ and six‐fold Ir), the TPD peak remained at the same temperature, but exhibits lower intensity. There are three possible explanations for this effect: 1) Either the CO binding energy is identical at the two‐ and five‐fold sites, 2) the two‐fold Ir diffuses to the five‐fold site with the CO molecule still attached during the TPD experiment, or 3) the five‐fold coordinated Ir does not adsorb CO at all. With increasing pre‐annealing temperature, the peak gets smaller and eventually disappears. This is because Ir atoms incorporated in subsurface six‐fold coordinated sites are inaccessible to CO. The continued lack of a CO desorption peak after annealing the sample to 960 K is evidence that the Ir nanoparticles observed in STM are encapsulated by iron oxide. This is attributable to the so‐called strong metal‐support interaction (SMSI), a well‐known phenomenon previously observed for Pt nanoparticles on both Fe_3_O_4_(001) and Fe_3_O_4_(111).^18^

**Figure 3:**
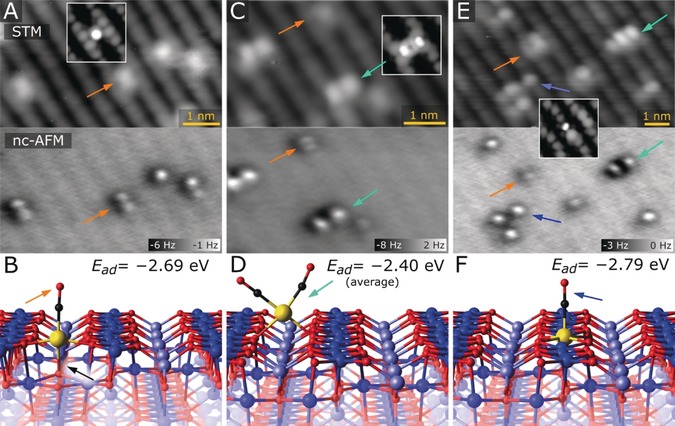
STM/ncAFM images of the Ir_1_/Fe_3_O_4_(001) model catalyst following exposure to CO at room temperature and corresponding DFT‐derived minimum‐energy structures. All of the images were acquired at 78 K using a CO‐functionalized tip. A) Ir_1_CO species (orange arrow) dominate when CO adsorbs on two‐fold‐coordinated Ir adatoms, and are imaged either as elongated protrusions between the surface Fe rows in STM (resolved into two distinct protrusions in ncAFM) and, in a minority of cases, as single bright protrusions. Note that the Fe rows of the support are imaged dark in the ncAFM images as these atoms weakly attract the CO tip. B) DFT‐derived minimum‐energy structure of an Ir_1_CO monocarbonyl. Note the additional bond (black arrow) that forms between the Ir adatom and an O atom in the subsurface layer, leading to a pseudo‐square‐planar environment. C) Ir_1_(CO)_2_ dicarbonyls appear with a significantly lower density, and are imaged as bright double protrusions perpendicular to the Fe rows (cyan arrow) in both STM and ncAFM. D) DFT‐derived minimum‐energy structure of an Ir_1_ dicarbonyl. Note the square planar environment of the Ir adatom. E) Heating the sample to 600 K leads to bright protrusions within the surface Fe rows (blue arrow). F) Minimum‐energy structures for the Ir_1_CO species formed at the five‐fold Ir atom calculated using DFT. Adsorbing the CO molecule completes an octahedral environment for the Ir atom. The insets shown in (A), (C), and (E) are STM simulations based on the structures shown in (B), (D), and (F), respectively. All images were taken with 1.5 V sample bias.

To investigate how CO interacts with the different Ir species, we performed further imaging experiments and DFT calculations. In Figure 3 A, we show STM and ncAFM measurements of the same area after a 0.08 ML Ir_1_/Fe_3_O_4_(001) sample was prepared as in Figure 1 A and then exposed to 3 L CO at room temperature (1 L=1.33×10^−6^ mbar⋅s). The images were acquired using a CO‐functionalized tip at 78 K. In STM, the majority species on this surface are protrusions between the surface Fe rows that are slightly elongated in the [110] direction (orange arrow). In ncAFM, the same species are resolved as two distinct protrusions. The bright contrast results from a repulsive interaction between the CO on the tip and the CO adsorbed on the Ir adatom. The DFT calculations show that adsorption of a single CO on the two‐fold Ir causes it to move towards the surface and form a weak bond to a subsurface O atom (Ir−O bond length 2.3 Å), which is offset from the Ir adatom along the [110] direction. The molecule tilts away from the surface normal to form an almost linear CO−Ir−subsurface‐O atom axis. Thus, the Ir has three bonds to lattice oxygen, and one to the CO molecule, and a pseudo‐square‐planar configuration. Two distinct protrusions are observed in ncAFM because the system switches rapidly between the two equivalent O atoms in the subsurface. This reorients the CO molecule to maintain the plane of the square, and a superposition of the two states is measured. The barrier for the switching process is calculated to be less than 0.1 eV. The distance between the protrusions observed in ncAFM appears larger than that calculated by DFT because the CO molecules adsorbed on the surface and tip repel and are then able to relax away from one another. In the configuration shown in Figure 3 B, the CO has a binding energy of −2.69 eV, while the Ir has a Bader charge of 0.83 (increased by 0.33 relative to the bare two‐fold adatom).

An alternative explanation for the features seen in Figure 3 A is that two CO molecules are adsorbed on each Ir atom, and oriented parallel to the Fe_row_ direction (creating a tetrahedral environment for the Ir). Such a configuration was calculated, and the adsorption energy was calculated to be −1.6 eV per molecule. However, the structure is unstable against the dicarbonyl configuration shown in Figure 3 D (−2.4 eV per CO molecule), in which the CO molecules creates a square planar environment for the Ir atom. Such species are imaged as bright double‐lobed protrusions with their axis perpendicular to the [110] direction, and frequently observed in STM/ncAFM images following room‐temperature CO exposure (Figure 3 C, cyan arrows). However, their density is consistently an order of magnitude lower than the density of IrCO monocarbonyls. Our DFT calculations suggest that the Ir atom in the dicarbonyl is two‐fold‐coordinated to the support and has a Bader charge of 0.77, which is close to that in the monocarbonyl. The average binding energy of −2.4 eV suggests that binding a second CO molecule is strongly favored thermodynamically, and thus should occur in the absence of kinetic limitations. We attribute the prevalence of IrCO species in our data to a combination of the low CO pressures used (*p*_CO_=1×10^−8^ mbar), and the repulsion between the IrCO and approaching CO molecules, which reduces the probability that Ir(CO)_2_ can be formed. At catalytically relevant CO pressures, this limitation will be quickly overcome and the Ir(CO)_2_ should dominate at room temperature for two‐fold‐coordinated Ir adatoms. To check that the features assigned as Ir(CO)_2_ and IrCO species are indeed related to Ir adatoms, we scanned the surface at 3.5 V to desorb the CO. This results in features identical to the as‐deposited Ir adatoms as judged by STM (Supporting Information, Figure S6), as expected.

When the CO‐exposed Ir_1_/Fe_3_O_4_(001) sample shown in Figure 3 A,C is annealed to 600 K and re‐imaged at 78 K, some Ir(CO)_2_ species remain, but the majority of protrusions are located within the surface Fe rows (see blue arrows in Figure 3 E). These species are very bright in ncAFM, consistent with strong repulsion between the CO on the tip and at the surface. This shows that the Ir species migrate to the five‐fold‐coordinated site in the surface prior to the desorption of the CO molecules. Additional evidence comes from STM experiments in which the CO was dosed on the two‐fold Ir_1_ at room temperature and the sample subsequently annealed such that Ir was incorporated into the five‐fold sites. After scanning a small area with high bias, the apparent height of the individual five‐fold Ir_1_ in the scanned area significantly increased, which is in line with STM simulations showing brighter contrast for bare five‐fold Ir_1_ than for five‐fold IrCO (Supporting Information, Figure S7). Thus, the TPD peak in Figure 2 B is always at the same temperature (610 K) because CO desorption always occurs from the five‐fold‐coordinated site. The peak decreases in intensity with pre‐annealing steps because some Ir is lost to the more stable six‐fold site, where it is inactive for CO adsorption.

Overall, our results show that Ir atoms can occupy multiple cation‐like sites on Fe_3_O_4_(001), and that the barriers between them are low enough that switching can occur at reaction temperatures relevant to single‐atom catalysis. Increasing the coordination from two‐, to five‐, and eventually six‐fold is energetically downhill, so once Ir is incorporated into the subsurface it will be difficult to recover. Previously, we have rationalized the preference of 3d transition metals (Ti, Mn, Co, and Ni11b) to occupy subsurface octahedral sites by analogy to Me_*x*_Fe_2−*x*_O_4_ ferrite compounds, where the dopant metal (Me) substitutes Fe in octahedral sites. We cannot apply this simple logic here, however, because Ir_*x*_Fe_2−*x*_O_4_ has not been synthesized and appears to be unstable. Nevertheless, Ir is octahedrally coordinated to O^2−^ anions in its stable oxide (IrO_2_, rutile structure), so the energetic cost of accommodating the large cation in the surface layer is not preclusive. Rather than diffusing far into the bulk at high temperatures, as the 3d metals do,11b the high cohesive energy of Ir (>7 eV per atom) comes to the fore and nanoparticles are formed. Our results show that incorporation into the oxide will deactivate an Ir‐based SAC well before thermal sintering into nanoparticles. Distinguishing between the five‐ and six‐fold species would be difficult by transmission electron microscopy because their position is identical with respect to the surrounding cation lattice viewed from above, and it is likely that incorporation occurs under reaction conditions.

In previous work, we have shown that the adsorption of CO at Pt and Pd adatoms accelerates thermal sintering,11c, 19 and this phenomenon has been observed in operando using TEM.^20^ Naively, one suspects that the metal‐oxide interaction weakens upon CO adsorption to conserve the bond order, and that this effect should be similar for all metals. However, not only do Ir atoms remain steadfastly in place during CO exposure, but adsorption of the molecule causes the Ir atom to interact more strongly with the surface. For example, the Ir atom shown in Figure 3 B is significantly closer to the surface than the bare adatom, with shorter Ir−O bonds and an additional bond to the subsurface. This leads to a significant increase in the Bader charge, suggesting a change in the oxidation state. This effect is important because the CO stretch frequency is typically used to measure the charge state of metal atoms in SAC, but is clearly not an innocent probe of the system, as reported previously for Au_1_/MgO.^21^ Moreover, the stretch frequency also depends critically on the local environment, and interpretation of experimental data requires theoretical support.6a, 22 XPS can be used to study the charge state of SACs without adsorbates,^23^ but the core‐level shift is also sensitive to the local structure, and either DFT or a well‐chosen reference sample is required to unravel the different contributions.

An interesting aspect of our findings is that from both the two‐fold and five‐fold Ir_1_ atoms CO desorbs at higher temperatures than from Ir(111) and Ir(100) surfaces.10a, 10c, 24 The most obvious difference between metal and oxide surfaces is the ionic binding, which makes the metal sites positively charged. While this introduces an electrostatic contribution to the binding of polar molecules, the CO dipole is small so this effect is expected to be weak. On metals, the CO binding strength is dominated by the extent to which the 2π antibonding states of the metal‐CO system are filled. One might expect, therefore, that a positively charged ion will have a higher d‐band, and thus exhibit stronger binding. This fits to Ir in principle, but is counter to the experience with Pt, however, where weaker back donation is thought to explain why positively charged adatoms bind CO more weakly than nanoparticles.1b, 6a It thus appears that there is no simple rule to predict how strongly CO will bind to a single‐atom catalyst, and that such systems must be studied on a case‐by‐case basis.

Our data suggests that strong CO binding in the Ir_1_/Fe_3_O_4_(001) system can be understood by analogy to Ir coordination complexes. In the as‐prepared state, the two‐fold‐coordinated adatom is stable, but cannot achieve a desirable linear bonding to oxygen due to the constraints of the surrounding lattice. Adsorbing CO allows the system to evolve to the stable square‐planar bonding environment, be it through the metastable monocarbonyl geometry or the dicarbonyl. Thus one can think of the two‐fold coordinated Ir adatom as a square planar Ir complex with two coordination vacancies. Similarly, the five‐fold Ir is an octahedral complex with one coordination vacancy, and filling this with CO stabilizes the system. Interestingly, we observe this latter interaction in the decrease in the apparent height observed in empty‐states STM images when the five‐fold Ir atoms adsorb CO. This is a purely electronic effect, which occurs because Ir states protruding from the surface within 1 eV above the Fermi level are involved in CO binding.

The analogy to coordination complexes is also borne out by the local magnetic moments obtained in our DFT calculations. The stable Ir dicarbonyl has a magnetic moment of 0, as expected for an ideal square planar Ir^I^ complex in a low‐spin d^8^ configuration. The monocarbonyl is unable to reach the ideal square‐planar geometry due to the constraints of the surface lattice, and its magnetic moment is an imperfect 0.3. Completion of the octahedral environment at the five‐fold Ir_1_ again leads to a magnetic moment of 0 in DFT, consistent with the low‐spin Ir^III^ state. In contrast, the bare two‐fold and five‐fold Ir adatoms have non‐zero magnetic moments of 1.11 and 0.28, respectively. We conclude that assigning oxidation states to the metal atoms in SAC makes sense only when compared to a suitable reference, such as a coordinatively saturated complex.

All of this shows that SAC systems are as much like homogeneous catalysts as they are heterogeneous catalysts. In the single‐atom limit, the bonding environment of the adatom will play a significant role in the strength with which reactants are bound, and in the number of reactants that can be bound at the single site. As such, simply synthesizing single‐atom variants of established nanoparticle catalysts is not an optimal approach, and experimental and theoretical screening is required to determine the metal/support combinations representing the best bet for a particular reaction. This, of course, requires the catalytic mechanisms at work to be firmly established. We show here that CO_2_ can be formed by extraction of O from the lattice, but that this process requires significant energy. Li et al.3a recently proposed that CO oxidation can occur with extremely low barriers through an OCOO intermediate adsorbed at the single site. In our view, such a process could be promoted by an adatom geometry possessing two coordination vacancies such as the two‐fold Ir demonstrated here. The five‐fold Ir, more stable at reaction temperatures, could not perform this function. Similar considerations hold for more complex reactions such as hydroformylation,^5^ which is typically performed by Rh complexes in solution and is a target for heterogenization by SAC. To mimic the mechanism of the complex requires that CO and an alkene are simultaneously coordinated at the metal center, which is something that only the two‐fold Ir could facilitate. While parallels to homogeneous catalysis clearly exist, O^2−^ is not a common ligand in such systems, and much needs to be learned about how the rigidity of the crystal lattice will affect the catalytic properties. In particular, it will be fascinating to see whether the strong binding enables the adaptive coordination reported recently for the Pd/g‐CN system.4b

Finally, it is interesting to note that CO adsorption stabilizes the Ir adatoms against incorporation into the substrate. The octahedral IrCO species remain stable on the surface until the CO desorbs at 610 K, but bare Ir_1_ atoms would incorporate into the subsurface six‐fold sites already at temperatures more than 100 K lower (Figure 2). This demonstrates that adding ligands can be a way to stabilize single atoms on surfaces, and one must wonder whether such a concept could be further utilized in SAC design. Again, this idea has parallels in homogeneous catalysis, where only a minority of the ligands are exchanged during a single reaction step, while the others fill the coordination vacancies of the metal center and thus keep the complex stable.

## Conclusion

Our results show that the choice of metal and the coordination environment have a significant effect on adsorption properties in SAC systems. Low‐coordination active sites allow the adsorption of multiple reactants, and novel reaction mechanisms analogous to homogeneous catalysis might be achievable. However, maintaining such sites will be difficult because higher coordination to the oxide will be strongly favored for most metals of interest. Finally, our work demonstrates that atomically resolved studies on well‐defined model SAC systems can play an important role in single‐atom catalysis research and are an ideal complement to state‐of‐the‐art theoretical calculations.

## Experimental and Theoretical Section

Natural Fe_3_O_4_(001) samples (SurfaceNet GmbH) were prepared in UHV by sputtering (1 keV Ar^+^ or Ne^+^, 10 min) and annealing (950 K, 10 min) cycles with every other annealing cycle done in oxygen background (*p* ${}_{O{}_{2}}$ =5×10^−7^ mbar, 20 min). Ir deposition was performed using e‐beam evaporators calibrated by temperature‐stabilized quartz microbalances (QCM). Three UHV setups were used in this study: A room‐temperature Omicron μSTM setup, a low temperature Omicron LT‐STM setup equipped with a q‐Plus sensor and a custom preamplifier,^25^ and a molecular beam setup specifically designed to study the surface chemistry of single‐crystal oxide samples. Full details of the latter setup are provided in reference^16^ and further details for all systems are provided in the Supporting Information. The Vienna ab initio simulation package (VASP)^26^ was used for all DFT calculations using the optB88‐DF^12^ van der Waals functional with an effective on‐site Coulomb repulsion term *U*_eff_=3.61 eV. Further theoretical details are contained within the Supporting Information.

## Conflict of interest

The authors declare no conflict of interest.

## Supporting information

As a service to our authors and readers, this journal provides supporting information supplied by the authors. Such materials are peer reviewed and may be re‐organized for online delivery, but are not copy‐edited or typeset. Technical support issues arising from supporting information (other than missing files) should be addressed to the authors.

Supplementary
