# Supplementary material for: Local Structure and Coordination Define Adsorption in a Model Ir1/Fe3O4 Single‐Atom Catalyst
Source: Angew Chem Int Ed Engl. 2019 Aug 19;58(39):13961–8. doi: 10.1002/anie.201907536 (PMC6790613; doi:10.1002/anie.201907536)
Supplement: Supplementary file 1 — Supplementary [file ANIE-58-13961-s001.pdf]

Angewandte Chemie
Eine Zeitschrift der Gesellschaft Deutscher Chemiker

Supporting Information

Local Structure and Coordination Define Adsorption in a Model Ir_1/Fe_3O_4 Single-Atom Catalyst

*Zdenek Jakub+, Jan Hulva+, Matthias Meier, Roland Bliem, Florian Kraushofer, Martin Setvin, Michael Schmid, Ulrike Diebold, Cesare Franchini, and Gareth S. Parkinson**

anie_201907536_sm_miscellaneous_information.pdf

Supporting information

Experimental and Theoretical Details

Three experimental setups were used throughout this study. The room-temperature STM images were acquired in a two-vessel UHV system (RT-STM) consisting of a preparation chamber (base pressure $<10^{-10}$ mbar) and an analysis chamber ($<7 \times 10^{-11}$ mbar) equipped with an Omicron µ-STM operated in constant-current mode. Electrochemically etched tungsten tips were sputter cleaned and conditioned by voltage pulses. The analysis chamber further includes commercial XPS, LEED and AES setups. The STM data were corrected for distortion and piezo creep as described in reference [1].

The low-temperature STM and ncAFM data were taken in a commercial Omicron LT-STM chamber equipped with an ncAFM head using q-Plus sensors [2] and a custom preamplifier [3]. The setup consists of a preparation chamber ($<10^{-10}$ mbar) and an analysis chamber ($<10^{-11}$ mbar). The tungsten tips used were electrochemically etched and further treated by in-situ self-sputtering [4] and scanning/pulsing on Cu(100). The CO molecules terminating the tips were spontaneously picked up during STM scanning and bias pulsing above IrCO species, similarly to previously reported CO-tip preparation on CO-covered Pt clusters [5]. The LT-STM images were taken in constant-current mode, the ncAFM images in constant-height mode.

The XPS and TPD data were collected in a UHV setup specifically designed for surface chemistry studies of single-crystal oxide samples. Full details of this experimental setup are given in reference [6]. Briefly, the chamber ($<8 \times 10^{-11}$ mbar) is equipped with a liquid-He flow cryostat, a home-built calibrated molecular beam source, a Hiden 3F PIC quadrupole mass spectrometer, a monochromated X-ray source with Al/Ag anode (SPECS XR50M, FOCUS 500), a hemispherical analyser (PHOIBOS 150), a focused ion gun with deflection unit (SPECS PU/IQE 12/38), and a rear view LEED optics (SPECS ErLEED). The molecular beam is formed by expansion of high-purity isotopically-labeled ^{13}CO (Linde Gas GmbH) through two differentially-pumped stages and precision-made orifices. This results in a well-defined beam spot with (3.60 ± 0.15) mm diameter. The sample was mounted on a Ta sample plate using Ta clips. A thin Au foil was placed between the sample and the sample plate to ensure good thermal contact, and the sample plate was attached to the cryostat via Ta rods and heated by direct current. The sample temperature was measured by a K-type thermocouple spot-welded to the sample plate.

In all setups, the natural $Fe_3O_4(001)$ samples (SurfaceNet GmbH) were prepared by sputtering (1 keV Ar^+ or Ne^+, 10 min) and annealing (950 K, 10 min) cycles with every other annealing cycle done in oxygen background ($p_{O_2} = 5 \times 10^{-7}$ mbar, 20 min). Metal deposition was done using e-beam evaporators (FOCUS GmbH) calibrated by temperature-stabilized quartz microbalances (QCM). A monolayer (ML) of adatoms is defined as 1 adatom per ($\sqrt{2}\times\sqrt{2}$)R45° surface unit cell of the $Fe_3O_4(001)$. In the RT-STM setup the calibration was checked by adatom counting; no systematic difference was observed.

The Vienna ab initio Simulation Package (VASP) [7] was used for all DFT calculations. The optB88-DF [8] van der Waals functional was used with an effective on-site Coulomb repulsion term U_{eff} = 3.61 eV for Fe to accurately model the oxide. The Projector Augmented Wave (PAW) method [9] describes the electron-ion interactions. The plane wave basis set cut-off energy was set to 550 eV. A Γ-centred k-

mesh of 5×5×1 was used for the bulk, $Fd\bar{3}m$, and (001) surface calculations (Γ-point only for 2×2 supercell). Calculations were performed with the experimental magnetite lattice parameter (a= 8.396 Å) using an asymmetric slab with 13 planes (5 fixed and 2 relaxed $Fe_{oct}O_2$ layers) and 14 Å vacuum. To avoid interaction between adsorbates, and to accurately model the experimental coverages, a (2 × 2) supercell was used (i.e. four times the (√2×√2)R45° reconstructed cell). Convergence is achieved when forces acting on ions become smaller than 0.02 eV/Å. STM simulations were performed using the software p4vasp and within the Tersoff-Harmann approach [10]. In all cases, the simulated images include states between the Fermi level (0 eV) and empty states at +1 eV with an isodensity of 0.0015 e/A^2.

Table S1: Binding energies, Bader charges, magnetic moments, and XPS core level shifts obtained from the structures shown in this work.

Configuration	Ir Binding energy (eV)	CO binding energy versus gas phase (eV)	Ir Bader charge (e)	Ir magnetic moment (μB, absolute value)	Ir 4f core level shift with respect to 2-fold Ir (eV)
2-fold Ir	– 5.25	-	0.50	1.11	-
2-fold Ir(CO)	-	– 2.69	0.83	0.34	0.69
2 fold Ir(CO)$_2$	-	– 2.40	0.77	0.05	1.98
5-fold Ir	– 6.22	-	1.40	0.28	1.10
5-fold Ir(CO)	-	– 2.79	1.45	0.04	2.39
6-fold Ir	– 7.14	-	1.40	0.12	1.91

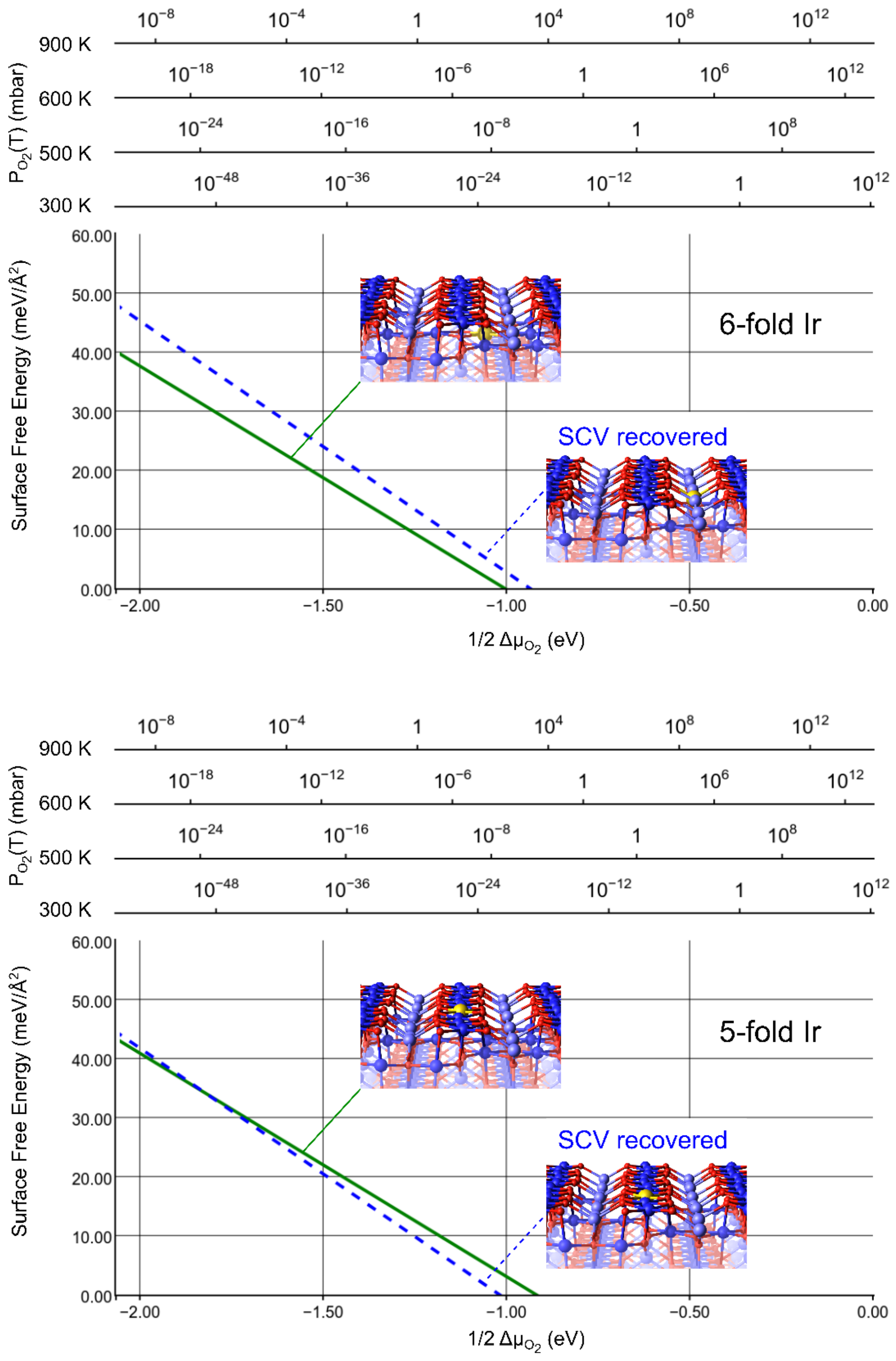

Figure S1: DFT-derived phase diagrams showing the change in the stability of the surfaces containing 6- and 5-fold incorporated Ir atoms depending whether the displaced Fe atom occupies one of the vacancies in the SCV reconstruction. The results suggest that 5-fold Ir is similarly stable with the subsurface defect at 10^{-12} mbar O_2 and 600 K, [11] whereas incorporation of Ir into 6-fold sites should be favoured with the additional Fe remaining in the surface region. We note that using the

experimental lattice parameter for Fe_3O_4 results in an under-estimation of the surface free energies. However, since we are primarily interested in the relative difference between the models, this offset is not important. At experimentally relevant O_2 pressures, the surface containing 5-fold Ir prefers to restore the SCV structure by diffusing an Fe atom into the bulk, which is possible at the considered temperatures [12]. This then allows the Ir to further incorporate to the 6-fold position more easily than if the displaced Fe atom remained in the 2nd octahedral layer.

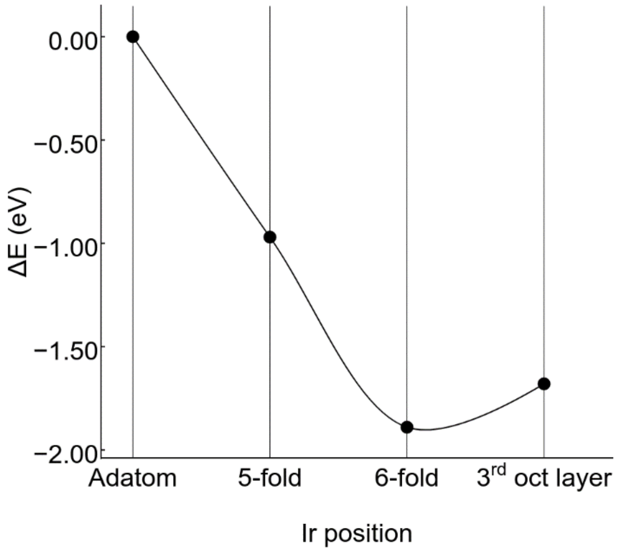

Figure S2: DFT-derived energies for the Ir/Fe_3O_4(001) system with the Ir occupying different sites. Energy is gained going from 2- to 5- to 6-fold coordination, but moving the Ir to deeper layers costs energy. Consequently, once Ir reaches the subsurface 6-fold site, it remains there.

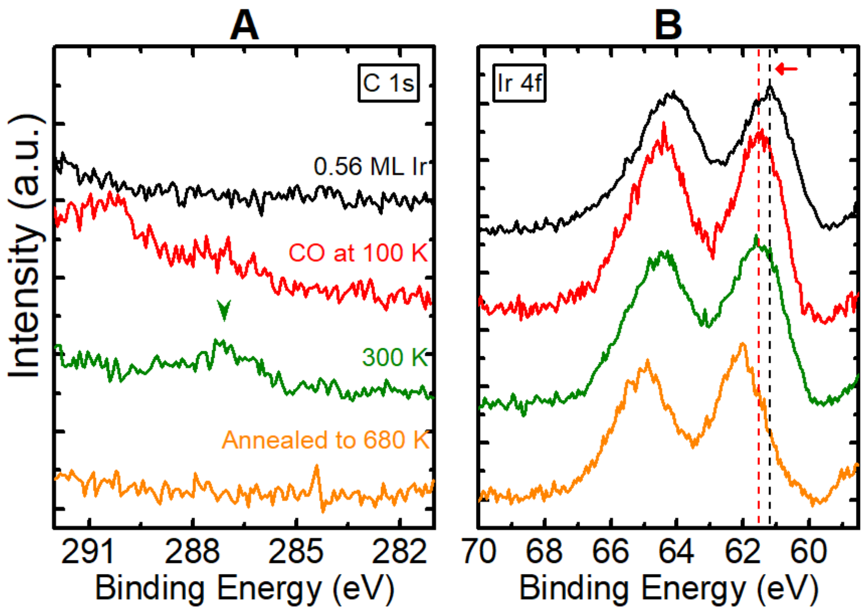

Figure S3: XPS of CO adsorbed on 0.6 ML Ir at 100 K and annealed to different temperatures. Excitation: monochromatized Al Kα; electron emission angle: 75° to the surface normal. **(A)** C 1s region. The peak corresponding to CO adsorbed on Ir is located at 287.1 eV. **(B)** Ir 4f region. Vertical lines mark positions of the peak after deposition (61.2 eV) and after saturation by CO at 100 K (61.5 eV).

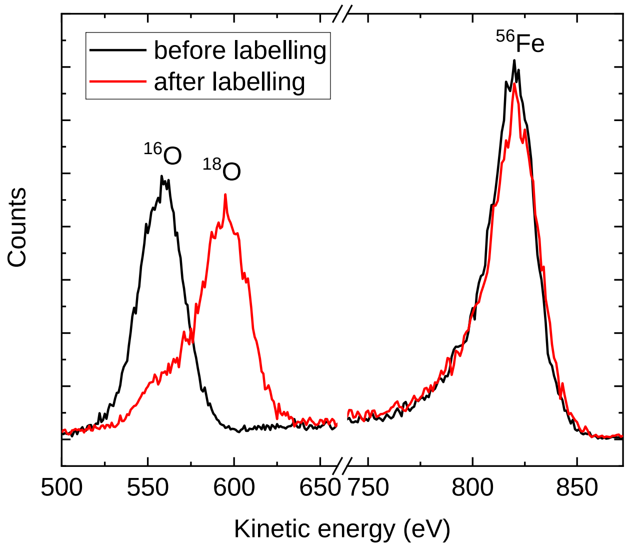

Figure S4: Low-energy ion scattering (LEIS) data (1 keV He^+) acquired from the as-prepared Fe_3O_4(001) surface (black) and after annealing in 1×10^{-6} mbar $^{18}O_2$ for 3 hours.

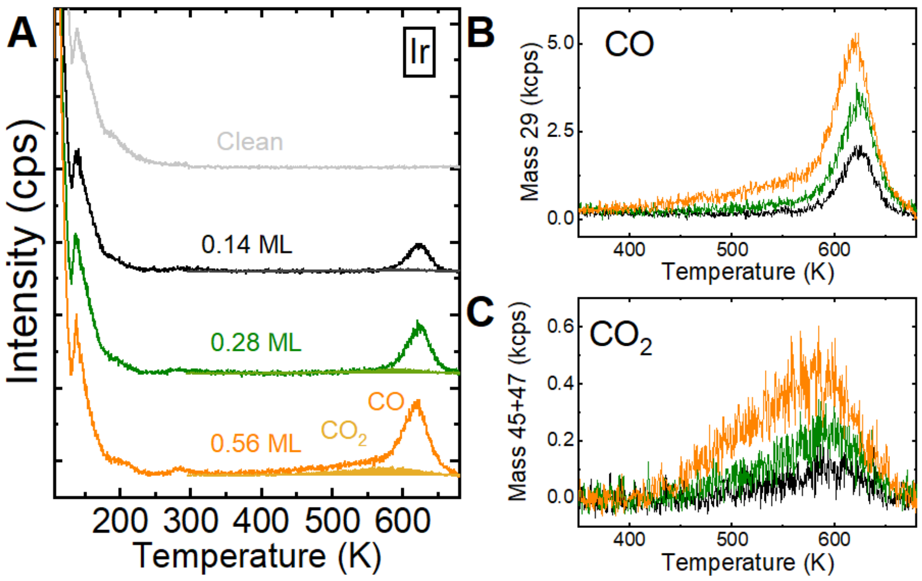

Figure S5: (A) CO TPD from different coverages of Ir on $Fe_3{}^{18}O_4$ (001). Thick curves show the CO desorption trace, and thin curves with the filled area underneath show the CO_2 signal. **(B)** Superimposed CO desorption curves between 300 K and 560 K. **(C)** CO_2 traces corresponding to (B).

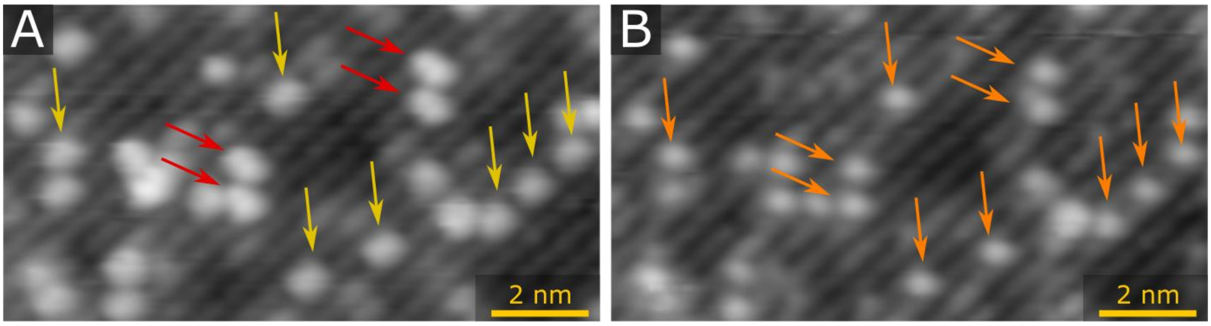

Figure S6: Room-temperature STM data that suggests the IrCO monocarbonyls and $Ir(CO)_2$ dicarbonyls form on similar 2-fold Ir_1 adatoms. Panels A and B show the same area scanned with the same parameters (U = 1.2 V, I = 0.3 nA) on a sample prepared by deposition of 0.2 ML Ir and 10 L CO. Before the acquisition of the image shown in panel B, the surface was scanned with 3.5 V bias. This leads to desorption of the CO from both the monocarbonyls (yellow arrows in A) and dicarbonyls (red arrows in A). In panel B, regular 2-fold Ir_1 adatoms (orange arrows) are resolved on positions corresponding to previous carbonyl species.

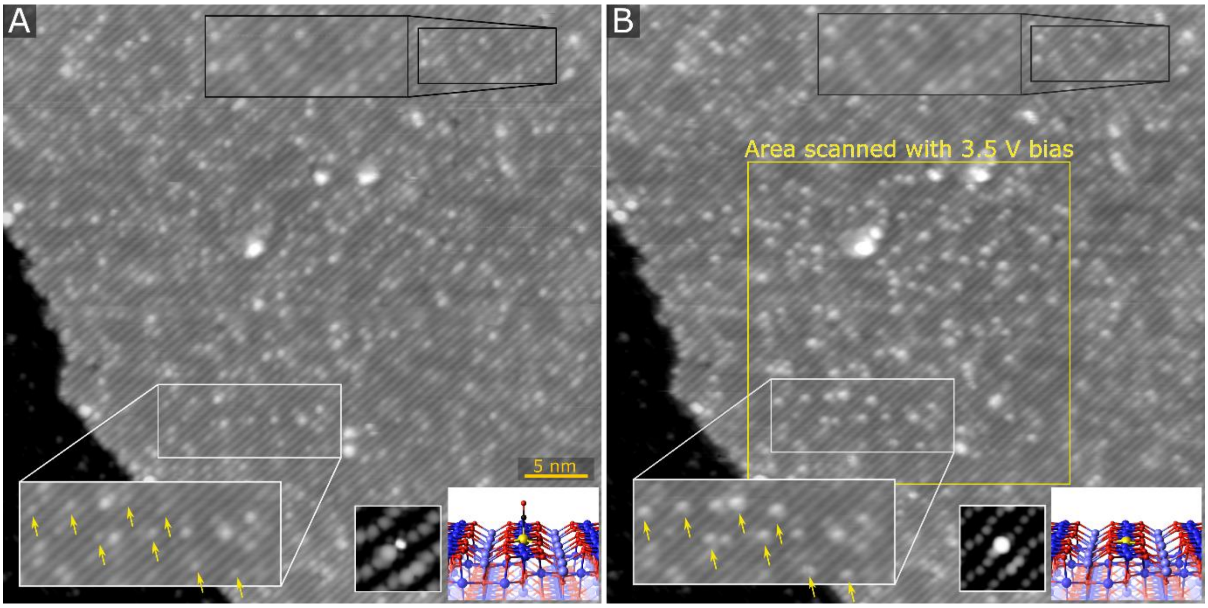

Figure S7: Additional evidence that CO remains adsorbed on the Ir_1 after it changes site to the incorporated 5-fold geometry. Panels A and B show room-temperature STM images acquired on the same area of a sample prepared by dosing 58 L CO on the surface with 0.2 ML 2-fold Ir_1. The sample was annealed to ca. 700 K to incorporate the resulting species into the 5-fold sites. Both images are scanned with the same parameters (U = 1.5 V, I = 0.3 nA), but before taking the image shown in B, a small area in the center (yellow square) was scanned with high bias (3.5 V). The in-the-row species in this square are significantly brighter than on the same area of panel A (the apparent height of the species marked with arrows is ca. 70 pm in panel A and ca. 110 pm in panel B). This is consistent with CO desorbing from the 5-fold Ir_1. The insets in bottom right of each panel show the DFT models and corresponding STM simulations. The insets in the top right of each panel show that this effect is not observed outside of the area scanned with high bias, which decisively rules out any significant tip change taking place between acquisition of the two images.

References

[1] J. I. J. Choi, W. Mayr-Schmölzer, F. Mittendorfer, J. Redinger, U. Diebold, M. Schmid, *J. Phys.: Condens. Matter* **2014**, *26*, 225003.

[2] F. J. Giessibl, *Rev. Sci. Instrum.* **2019**, *90*, 011101.

[3] F. Huber, F. J. Giessibl, *Rev. Sci. Instrum.* **2017**, *88*, 073702.

[4] M. Setvín, J. Javorský, D. Turčinková, I. Matolínová, P. Sobotík, P. Kocán, I. Ošťádal, *Ultramicroscopy* **2012**, *113*, 152-157.

[5] a) M. Meier, J. Hulva, Z. Jakub, J. Pavelec, M. Setvin, R. Bliem, M. Schmid, U. Diebold, C. Franchini, G. S. Parkinson, *Proc. Natl. Acad. Sci. U. S. A.* **2018**, *115*, E5642-E5650; b) Z. Jakub, F. Kraushofer, M. Bichler, J. Balajka, J. Hulva, J. Pavelec, I. Sokolović, M. Müllner, M. Setvin, M. Schmid, U. Diebold, P. Blaha, G. S. Parkinson, *ACS Energy Lett.* **2019**, *4*, 390-396.

[6] J. Pavelec, J. Hulva, D. Halwidl, R. Bliem, O. Gamba, Z. Jakub, F. Brunbauer, M. Schmid, U. Diebold, G. S. Parkinson, *J. Chem. Phys.* **2017**, *146*, 014701.

[7] a) G. Kresse, J. Furthmüller, *Comput. Mater. Sci.* **1996**, *6*, 15-50; b) G. Kresse, J. Hafner, *Phys. Rev. B* **1993**, *48*, 13115-13118.
[8] a) M. Dion, H. Rydberg, E. Schröder, D. C. Langreth, B. I. Lundqvist, *Phys. Rev. Lett.* **2004**, *92*, 246401; b) J. Klimeš, D. R. Bowler, A. Michaelides, *J. Phys.: Condens. Matter* **2009**, *22*, 022201; c) K. Lee, É. D. Murray, L. Kong, B. I. Lundqvist, D. C. Langreth, *Phys. Rev. B* **2010**, *82*, 081101; d) G. Román-Pérez, J. M. Soler, *Phys. Rev. Lett.* **2009**, *103*, 096102.
[9] a) P. E. Blöchl, *Phys. Rev. B* **1994**, *50*, 17953-17979; b) G. Kresse, D. Joubert, *Phys. Rev. B* **1999**, *59*, 1758-1775.
[10] J. Tersoff, D. R. Hamann, *Phys. Rev. Lett.* **1983**, *50*, 1998-2001.
[11] a) M. W. Chase, *J. Phys. Chem. Ref. Data* **1998**, *Monograph 9*, 1-1951; b) K. Reuter, M. Scheffler, *Phys. Rev. B* **2001**, *65*, 035406; c) K. Reuter, M. Scheffler, *Phys. Rev. B* **2007**, *75*, 049901.
[12] O. Gamba, J. Hulva, J. Pavelec, R. Bliem, M. Schmid, U. Diebold, G. S. Parkinson, *Top. Catal.* **2017**, *60*, 420-430.
